# Supplementary material for: Clinical Outcomes and Prognostic Factors for Extramammary Paget’s Disease Treated with Radiation Therapy: A Multi-Institutional Observational Study
Source: Cancers (Basel). 2025 Apr 29;17(9):1507. doi: 10.3390/cancers17091507 (PMC12070834; doi:10.3390/cancers17091507)
Supplement: Supplementary file 1 [file cancers-17-01507-s001.zip › cancers-3557006-supplementary.pdf]

Supplementary Table S1. The initial sites of distant metastases.

**Sites**	***n* = 20**
Lungs	4 (20%)
Bone	4 (20%)
Para-aortic LN	2 (10%)
Brain	1 (5%)
Liver	1 (5%)
Bone and liver	1 (5%)
Liver and spleen	1 (5%)
Liver and mediastinal LN	1 (5%)
Lungs, bone, and abdominal cavity	2 (10%)
Pleura, abdominal cavity, and subcutaneously	1 (5%)
Lungs, bone, and bladder	1 (5%)
Lungs, bone, liver, and pleura	1 (5%)

LN = lymph node.
